# Language and Cognitive Development in Deaf Children

**DOI:** 10.3390/bs16020169

**Published:** 2026-01-26

**Authors:** Gary Morgan, Mario Figueroa, Beatriz de Diego-Lázaro

**Affiliations:** 1 Department of Psychology and Educational Science, Universitat Oberta de Catalunya, Rambla del Poblenou, 156, 08018 Barcelona, Spain; 2 Department of Basic, Developmental and Educational Psychology, School of Psychology, Edifici B, Autonomous University of Barcelona, Cerdanyola del Vallès (Bellaterra), 08193 Barcelona, Spain; mario.figueroa@uab.cat; 3 Department of Cognition, Development and Educational Psychology, University of Barcelona, Passeig de la Vall d’Hebron, 171, 08035 Barcelona, Spain; bdediego@ub.edu; 4 Institute of Neurosciences, University of Barcelona, Passeig de la Vall d’Hebron, 171, 08035 Barcelona, Spain; 5 Department of Educational Psychology, Universidad de Valladolid, 400005 Segovia, Spain

## 1. Introduction

In this editorial, we introduce the topic of the Special Issue: the effects of deafness on language and cognitive development. We then outline current debates concerning this area of research and summarise the papers included in the Special Issue. We conclude with areas we consider valuable for future research. Over several decades, the profiles of Deaf and Hard of Hearing (DHH) children have been extensively documented, regarding perceptual abilities, social cognition, language, executive functions (EFs), and academic achievement. The large variability in outcomes observed in research studies has been linked to several factors, including family hearing status (deaf or hearing parents), the availability of hearing technology, and access to appropriate and early interventions. Early exposure to signed language, combined with inclusive educational support, significantly improves communication and developmental outcomes in deaf children (Yoshinaga-Itano et al., 2024).

Beyond deafness, there is significant current interest in identifying the factors that cause variability in development among the wider population of hearing children. Thus, an exploration of how and why deafness influences development has far-reaching theoretical implications for understanding how all children develop language. Cognitive abilities are related to language development and play a role in the variability observed across all children in how fast and well they learn to communicate. For example, basic attentional control during the first year supports the segmentation of the speech stream and facilitates the beginning of intentional communication (Salley et al., 2016). Studying DHH children allows us to explore broader developmental questions and observe how different factors—such as cognitive elements—impact development. Examples include the roles of deafness and language on EFs where studies on children who are DHH show that EF delays are reduced when they have early access to language—such as a signed language from birth or early cochlear implant (Nicastri et al., 2021). However, establishing a theoretical model or defining a specific mechanism to explain these complex interactions remains challenging.

Research into DHH children also aims to inform evidence-based practice. Unfortunately, much educational and clinical practice lacks evidence supporting teaching methods or demonstrating that certain therapy approaches make a significant developmental difference (Knoors & Marschark, 2018). Translating academic research findings into practical, real-world professional (e.g., educational, clinical) settings is challenging due to the varied and sometimes conflicting results of studies, the heterogeneity of the population included in samples, and the values and assumptions of the researchers and professionals involved. These dynamics reflect a field in flux, with ongoing debate about the best approaches to support DHH individuals in a world with evolving technology and shifting cultural perspectives.

## 2. Characteristics of Childhood Deafness

Around 5–10% of DHH children are born to DHH parents who generally can provide immediately accessible social interaction through signed language. Ninety to ninety-five percent of DHH infants, however, are born to hearing parents, with no experience of deafness or signed language. Therefore, the large majority of DHH children with hearing parents are exposed to and learn spoken language and varying amounts of signs. In these families, a particular early challenge is the establishment of communicative routines and language development. At the same time during this early period, hearing technology is being implemented and the infant is becoming accustomed to this artificial way of perceiving sounds. This means that the population of DHH children varies greatly in how well they learn spoken language, as most begin their first years with language learning delays, which diminish with age. Since the advent of early hearing detection and intervention coupled with improvements in hearing technology, especially cochlear implants (CIs), the majority of school-age DHH children perform within the average range on standardised language measures (Tomblin et al., 2020). Despite this, approximately 30% of DHH children still experience persistent language difficulties (Bruijnzeel et al., 2016) and often with the more advanced parts of language. These parts include complex grammar and pragmatics, comprising the social use of language in different contexts. Good language skills support the school experience, so any delays can lead to literacy difficulties, behavioural challenges, and reduced school functioning (McKean et al., 2017).

## 3. Current Debates Concerning This Topic

Research on deafness has evolved rapidly over the past 20–25 years, driven primarily by technological advancements like CIs and a growing acknowledgment of the cultural model of deafness alongside the traditional medical or deficit model.

### Complexity of Participant Groups

DHH children are made up of sub-groups who have different patterns of strengths and weaknesses related to the development of language and cognitive skills. In the past, there was a tendency to mix DHH children with different characteristics (e.g. severity of deafness) into a single group. More recently, papers have presented more nuanced descriptions of participant demographics. Today, the fact that DHH children are not considered to all be one group is seen as important, as the groupings produce different types of outcomes. We see across the literature, and even in the small number of papers in this Special Issue, that differing results are obtained depending on which sub-groups of DHH children are focused on.

The diverse range of communication modalities, ages of implantation, and family backgrounds (e.g., DHH children of DHH parents who use CIs) makes creating “clean” research groups difficult. Studies often face the dilemma of reflecting this “messy” reality or seeking homogenous groups, which may create a false reality that does not represent the broader DHH population. There are notable differences in how researchers interpret data, often influenced by their underlying model of deafness. Some studies may attribute all positive language outcomes to signing, for example, even when children have significant spoken language input, while others focus solely on spoken language gains in contexts of bimodalism. The complexity of mixed modalities and varied input makes isolating specific factors challenging. In addition to this variability, many DHH children come from immigrant families who speak an oral language other than the majority language, resulting in bilingual or trilingual DHH children (e.g., in North America: English, Spanish, and a signed language). In addition the prevalence of deafness in developing countries is higher than in Western European countries (1–5: 1000 births per year; up to 19–24: 1000 in developing countries). Multilingualism is an additional challenge for assessments, interventions, and research protocols. Unsurprisingly, multilingual DHH children are often excluded from research studies. Most of the published studies have been conducted on DHH children from Western countries (mostly white and English speakers), which are not representative of the worldwide DHH population.

## 4. Summary of the Papers Included in the Special Issue

There are three main areas covered in this Special Issue: (i) language development, (ii) cognitive and social-cognitive development, and (iii) literacy development.

### 4.1. Language Development

Most research on DHH children focuses on speech and spoken language. Speech discrimination is a foundational auditory skill but the inability to properly categorise speech sounds based on audition alone in childhood can increase the risk of spoken language and literacy delays later in life. The paper by **Walker and colleagues** compares speech discrimination abilities between children who are DHH and children with typical hearing (TH) aged 9 to 36 months. The researchers were careful when testing children with hearing aids in aided and unaided conditions, and each phoneme contrast was tested twice to control for learning effects. When speech discrimination abilities were compared between both groups, there were no statistical differences in performance on stop consonant discrimination, but a significant statistical difference was observed for fricative discrimination performance. There was also an effect of listening fatigue on speech discrimination. It is well documented that DHH children require higher levels of attention and concentration to process auditory information compared to TH children. Regarding an under-researched area, the next paper by **Hilker et al.** looked at filled pauses in discourse contexts in 10-year-old children who are DHH and children with TH. Filled pauses are considered to be reflections of linguistic processes (e.g., lexical retrieval, speech planning and execution). “Uh” may be a self-directed cue for when a speaker needs more time to retrieve lexical–semantic representations, whereas “um” serves as a listener-directed, pragmatic cue. The use of filled pauses has not been previously examined in children who are DHH. Their results indicate that “um” and “uh” were used more often in conversational samples compared to other types of discourse. DHH children did not differ in their use of “um” relative to TH children; however, they produced “uh” more often than TH children, suggesting that they may have difficulty retrieving lexical–semantic items during ongoing speech. This information may be useful for interventionists collecting language samples during assessment.

### 4.2. Cognitive and Social-Cognitive Development

This complex topic has the potential to reveal insights into wider child development as DHH children can highlight how language and wider cognitive systems interact in development. Research on language development in DHH children has largely focused on single-modality contexts (spoken or signed language), leaving the dynamics of simultaneous signed and spoken language acquisition (bimodalism) underexplored. The paper by **Lillo-Martin and colleagues** presents three case studies of DHH children with hearing parents learning American Sign Language (ASL) and English simultaneously. The children were exposed to native English from their parents and a learner’s version of ASL (as their parents were learning ASL at the same time as their children). The study looked at different aspects of language and cognitive development and asked whether reduced early access to language input led to any delays. The study found strong language outcomes in the three children and, consequently, age-appropriate cognitive development. These results are consistent with the conclusion that novice signer parents can support their children’s development as ASL–English bilinguals, establishing a strong foundation for further cognitive and linguistic growth.

As mentioned previously, EFs have received great interest recently in studies of DHH children, but results have been mixed with regard to the sub-sample of children tested. Previous studies that included the group of 95% of DHH children with hearing parents not exposed to a signed language demonstrated significantly poorer performances in EF, and this is often related to language skills. The paper by **Terhune-Cotter and Dye** assessed serial working memory (WM) using the n-back task. They carried out a longitudinal study on a large sample of DHH children who acquired ASL as their first language (N = 103). Their results showed a significant growth in EF skills across the 7–13-year-old age range. Furthermore, children with early access to ASL from their DHH caregivers demonstrated faster WM growth, and this was mediated by ASL receptive skills. The data suggest the important role of early access to perceivable natural language in promoting typical WM growth. The authors also highlight whether variability is driven by acquisition-related corollaries, such as parent–child interactions and maternal stress.

The link between language development and social cognition in DHH children has become an area of considerable scholarly interest. **Martin and colleagues** carried out a systematic review on the topic of Theory of Mind (ToM) development in DHH individuals. During the last three decades, ToM development has been studied extensively in DHH individuals and performances compared to the TH population. Given the advances in the early diagnosis of deafness, interventions, and hearing devices over this period, variations in task performance among DHH participants might have decreased. The two take-away messages from this systematic review were that most ToM studies find a range of delays and that ToM variance across DHH children is related to several factors, but especially to quality of early interactions and early exposure to both signed and spoken language. Secondly, there has been a narrow focus of research on false beliefs, compared to the range of ToM abilities outlined at the outset of social-cognitive research.

The second paper on the link between language and social cognition concerned a study by **Intxaustegi et al.**, which focused on hidden emotion understanding and its relationship with mentalistic verbs. Hidden emotions involve situations in which individuals deliberately express an emotion that is different from what they genuinely feel and is a key skill in ToM development. This ability allows children to reason about discrepancies between internal emotional states and external expressions. This is closely tied to language development, particularly to vocabulary related to mental states, which supports complex emotional reasoning. DHH children aged 7 to 12 years raised in oral language environments completed a hidden emotions task. TH children outperformed their DHH peers in understanding hidden emotions, and this was attributed to language use. Specifically, children’s spontaneous use of cognitive verbs in their explanations (e.g., think, know) predicted task performance. These findings underscore the importance of early and accessible language exposure in supporting the emotional and social-cognitive development of DHH children.

As described in Section 1, an important factor in DHH children’s development is the role of the surrounding family. Continuing to look at social-cognitive skills, the study reported by **Ketelaar and colleagues** explored Parental Mental State Talk (MST) and its association with social-emotional development. This paper bases its analysis of ToM development on the idea that early parent–child interactions are crucial for children’s social-emotional development. MST—language directed to children referring to thoughts, feelings, and intentions—may be reduced in hearing parents of DHH children. However, the results showed that parents adjusted MST complexity based on children’s age, but not on audiological characteristics. Parental and child MST were associated, though not linked to socio-emotional performance, thus suggesting a far-from-simple relationship.

### 4.3. Literacy Development

The final area covered in this Special Issue concerns the relationship between print literacy, language, and cognitive development. The study by **Wolbers et al.** investigated variability in language and literacy outcomes in 368 DHH elementary students, focusing on reading and writing performance and their connections with demographic and language variables. Their results indicated wide variability in reading and writing performance and a strong positive correlation between them, suggesting interconnected development of literacy skills. Language proficiency (in ASL and/or spoken English) and phonological knowledge (fingerspelling and/or spoken modality) explained 55–63% of the variance in literacy outcomes, highlighting the need for early accessible language exposure and responsive literacy instruction.

The final paper is a scoping review of literacy interventions using signed languages. The review, carried out by **Dostal et al.**, indicates that integrating signed language into literacy instruction enhances language access and supports literacy learning. The results highlight the importance of responsive, multimodal instruction and indicate the need for further research to fill identified gaps in how the use of signed language could be implemented into teaching strategies.

## 5. Overall Conclusions and Future Directions

In this Special Issue, we address a range of topics on DHH children’s language and cognitive development. In summary, across the papers collected here, there is a high amount of variability in the outcomes reported for different groups of children. In several areas of cognitive development, there is a complex relationship between early access to spoken and signed language, successful language development, and wider abilities. Despite advances in CI and intervention, differences remain, especially in higher cognitive areas such as social cognition and literacy. The two main areas we consider valuable for future research studies are the following: first, more research on DHH children who use and develop a mixture of sign and speech and its impact on related cognitive skills; and second, researchers should aim to provide more concrete and usable evidence for future language and educational practices.
